# Single-cell RNA-sequencing reveals cellular heterogeneity and immune microenvironment characteristics between ocular adnexal mucosa-associated lymphoid lymphoma and IgG4−related ophthalmic disease

**DOI:** 10.3389/fimmu.2025.1508559

**Published:** 2025-02-26

**Authors:** Yu Yang, Yujiao Wang, Xuelian Jin, Weimin He

**Affiliations:** ^1^ Department of Ophthalmology, West China Hospital, Sichuan University, Chengdu, Sichuan, China; ^2^ Department of Ophthalmology, Sichuan Academy of Medical Sciences & Sichuan Provincial People’s Hospital, University of Electronic Science and Technology of China, Chengdu, Sichuan, China; ^3^ Department of Hematology, West China Hospital of Sichuan University, Chengdu, Sichuan, China

**Keywords:** ocular adnexal, mucosa-associated lymphoid tissue lymphoma, IgG4-related ophthalmic disease, single-cell RNA sequencing, immune microenvironment, cellular heterogeneity

## Abstract

**Introduction:**

The molecular pathogenesis of ocular adnexal mucosa-associated lymphoid tissue (MALT) lymphoma and IgG4-related ophthalmic disease (IgG4-ROD) remains incompletely understood. Differentiating between the two diseases is vital given that the diagnostic evaluation and treatment approaches can vary significantly; this difficulty in distinction is exacerbated by the absence of specific biomarkers. This study aimed to investigate the differences between these two diseases based on their cellular composition, transcriptional heterogeneity, and the immune microenvironment using single-cell RNA transcriptional sequencing (scRNA-seq) technology.

**Methods:**

We collected orbital lacrimal gland region tissue samples from three patients with MALT lymphoma and another three with IgG4-ROD and performed single-cell sequencing experiments. Subsequently, we conducted bioinformatics analyses, including cell subpopulation segmentation and inter-group comparison, tumor cell identification, functional enrichment analysis, and pseudotime trajectory analysis. Furthermore, we analyzed the cellular communication between tumor B-cell and T-cell subsets within the immune microenvironment of MALT lymphoma tissues. We performed immunofluorescence assays to verify the co-expression of receptor-ligand pairs.

**Results:**

A total of six major cell subpopulations were identified, with B-cells and T-cells being the predominant cell types. All B-cell subpopulations in MALT lymphomas are malignant, exhibiting significant intratumoral and intertumoral heterogeneity. Reclustering of the T-cell subpopulation identified five major T-cell subpopulations. Pseudotime analysis revealed that CD4+ naive T-cells in MALT lymphoma patients were highly likely to differentiate into follicular helper T-cells, whereas, in IgG4-ROD patients, CD4+ naive T-cells were highly likely to differentiate into regulatory T-cells. Intercellular communication analysis revealed that the CD27-CD70 immune checkpoint receptor−ligand pair and CXCL13-CXCR5 chemokine receptor−ligand pair were significantly upregulated between malignant B-cells and T-cells subpopulations.

**Conclusion:**

This study is the first to conduct a comparative single-cell transcriptome sequencing analysis of ocular adnexal MALT lymphoma and IgG4-ROD. Our results reveal the cellular composition, key pathways, and critical immune microenvironment implicated in the development of these two diseases. These findings provide important insights into the pathogenesis of these two diseases and highlight the differences between them.

## Introduction

Ocular adnexal mucosa-associated lymphoid tissue (MALT) lymphoma is a low-grade non-Hodgkin B-cell lymphoma affecting the MALT. It is characterized by a relatively indolent clinical progression and represents the most prevalent histopathological subtype of ocular adnexal lymphoma (1–3). Advancements in tumor detection technologies, alongside a shift in environmental factors and lifestyle habits, have contributed to the observed rise in the incidence of ocular adnexal MALT lymphoma. Prognosis is relatively favorable in most patients; however, a small proportion of the patients may experience a relapse and subsequent cancer progression, with a possibility of the condition transforming into high-grade aggressive lymphomas (4–8). While the etiology and pathogenesis of ocular adnexal MALT lymphoma remain incompletely understood, research indicates that both genetic factors and immune dysregulation significantly contribute to the development of this disease (9–11). Currently, an increasing number of studies have indicated that the occurrence and development of ocular adnexal MALT lymphoma are closely associated with autoimmune diseases, particularly IgG4-ROD (12–14).

Notably, IgG4-ROD is a chronic inflammatory autoimmune disease characterized by elevated serum IgG4 levels and infiltration of IgG4-positive plasma cells. Both IgG4-ROD and MALT lymphoma are lympho-proliferative diseases (15, 16), characterized by similar clinical and pathological features, and can occur either concomitantly or sequentially in a patient; indicating that IgG4-ROD is a potential precursor to MALT lymphoma (14, 17). Despite similarities in their clinical presentations, these diseases exhibit significant variability in terms of their benign or malignant nature, molecular characteristics, treatment modalities, and prognosis (18–21). Consequently, clinical distinction between these diseases is very crucial for their effective management.

Single-cell RNA sequencing (scRNA-seq) is a cutting-edge sequencing technique that enables the extraction of genetic information from individual cells within tissues. Notably, this technique enables the acquisition of gene sequences, transcripts, proteins, and epigenetic information of specific cell subpopulations, thereby facilitating analysis of genetic and protein differences between cell subpopulations (22). Currently, there are no scRNA-seq studies on the differences between ocular adnexal MALT lymphoma and IgG4-ROD. Consequently, this study focuses on using scRNA-seq to highlight the differences between these two diseases in terms of cell composition, transcriptional heterogeneity, and the immune microenvironment.

## Materials and methods

### Sample collection

This study used six tissue samples: three MALT lymphoma and three IgG4-ROD samples from the orbital lacrimal region. These tissue samples were obtained from six patients within the Department of Ophthalmology at West China Hospital, Sichuan University. The clinical characteristics of the six patients are summarized in **Table 1**. Fresh pathological tissues were immediately excised and a portion approximately the size of soybean was preserved in GEXSCOPE tissue preservation solution at 2–8°C for subsequent single-cell analysis. The remaining tissue was promptly immersed in a tissue fixative followed by paraffin embedding. All patients were definitively diagnosed using pathological examination. The use of the study samples was approved by the Ethics Review Committee of West China Hospital, Sichuan University [Approval No: 2022 Review (47)]; additionally, informed consents were obtained from all patients.

**Table 1:** Clinicopathological characteristics of included patients.

	Sex	Age	Eye	Course	Involvement Sites	Pathological Diagnosis
**Patient 1**	Male	62	Left	12	Orbit, Lacrimal gland	IgG4-ROD
**Patient 2**	Female	68	Left	60	Orbit, Lacrimal gland	IgG4-ROD
**Patient 3**	Female	55	Right	24	Orbit, Lacrimal gland	IgG4-ROD
**Patient 4**	Male	54	Right	12	Orbit, Lacrimal gland	MALT
**Patient 5**	male	74	Left	6	Orbit, Lacrimal gland	MALT
**Patient 6**	Female	67	Right	7	Orbit, Lacrimal gland	MALT

### Single-cell suspension preparation

The samples were washed three times with Hanks’ balanced salt solution (HBSS, Gibco, Cat. No.14025-076) and sectioned into a thickness of 1–2 mm. The tissue debris was subsequently digested with 2 ml of GEXSCOPE Tissue Dissociation Solution (Singleron) at 37°C for 15 min in a 15 ml centrifuge tube (Falcon, Cat. No. 352095) with continuous agitation. The cell suspension was filtered through 40-micron sterile strainers (Falcon, Cat. No. 352340) and centrifuged (Eppendorf, 5810R) at 300 × g for 5 minutes. The supernatant was aspirated and the cell pellets resuspended in 1 ml of PBS (HyClone, Cat. No.SA30256.01). To eliminate the red blood cells, often a significant portion of the preparation, 2 mL RBC lysis buffer (Roche, Cat. No. 11 814 389 001) was added to the cell suspension according to the manufacturer’s protocol. The cells were centrifuged at 500 × g for 5 min in a microfuge at 15-25°C and resuspended in 1mL PBS. A sample from the cell mixture was stained with trypan blue (Bio-Rad, Cat. No. 1450013) and diluted to a concentration of 1×10^5^ cells/mL. Cell viability was examined microscopically (Nikon, ECLIPSE Ts2) and subsequent cell processing was conducted once viability exceeded 80%.

### Single-cell RNA sequencing

Single-cell suspensions at a density of 1×10^5^ cells/mL were prepared using PBS. Single-cell suspensions were then loaded onto microfluidic devices and scRNA-seq libraries were constructed according to the Singleron GEXSCOPE protocol using the GEXSCOPE Single-Cell RNA Library Kit (Singleron Biotechnologies). This process included cell lysis, mRNA capture, labeling of cells with barcodes and mRNAs with UMIs, reverse transcription of mRNAs into cDNA and amplification, and fragmentation of the cDNA. Individual libraries were diluted to a concentration of 4 nM and subsequently pooled for sequencing. The pooled libraries were then sequenced using an Illumina HiSeq X with 150 bp paired-end reads.

### Primary analysis of raw read data

An internal pipeline was used to generate gene expression matrices through analysis of the raw reads from the scRNA-seq data. The process involved the following steps: raw reads were first processed with fastQC and fastp to remove low-quality reads; then cutadapt was used trim poly-A tail and adapter sequences. Subsequently, the cell barcode and UMI were extracted. The reads were then mapped to the reference genome GRCh38 using the STAR (v2.5.3a) software. The UMI and gene counts of each cell were conducted using the Counts (v1.6.2) software, and the results were used to generate gene expression matrix files for subsequent analysis.

### Quality control, dimension reduction, and clustering

Due to the differences in sample sizes, we performed data normalization and batch effect correction. Before analyses, cells were filtered based on the following criteria: UMI counts below 30,000, gene counts between 200 and 5,000, exclusion of cells with over 20% mitochondrial content. After filtering, dimension reduction and clustering were conducted using the functions from Seurat v2.3. Subsequently, we used the normalize and scale Data functions to normalize and scale all gene expression, followed by the selection of the top 2,000 variable genes using the Find Variable Features function. These variables were identified for further PCA, with the top 20 principal components used to classify the cells into multiple clusters using FindClusters. The batch effect between samples was removed via Harnomy. Finally, visualization of the cells was conducted in a two-dimensional space using the UMAP algorithm.

### Differentially expressed gene analysis

To identify the differentially expressed genes (DEGs), we used the Seurat FindMarkers function based on the Wilcox likelihood ratio test with default parameters. Genes were considered differentially expressed if they were identified in over 10% of the cells in a cluster and exhibited an average log (fold change) value greater than 0.25. Cell type annotation for each cluster was conducted by integrating the expression of canonical markers identified in the DEGs with existing knowledge from the literature. The expression of these markers for each cell type was visualized using heatmaps, dot plots, and violin plots, generated with the Seurat DoHeatmap, DotPlot, Vlnplot functions. DoubleT-cells, which expressed markers characteristics of multiple cell types, were manually identified and removed from the analysis.

Examinations of the potential roles of the DEGs were conducted through Kyoto Encyclopedia of Genes and Genomes (KEGG) analyses using the “clusterProfiler” R package. Significantly enriched pathways involved those with p_adj values < 0.05.

### Trajectory analysis

Pseudotime trajectory analysis was performed using Monocle2 to map the differentiation of cell subtypes in CD4+ T-cells. We identified variable genes and ordered the cells onto a pseudotime trajectory based on the union of highly variable genes observed in all cells. Gene expression dynamics associated with cell state transitions can be inferred by ordering the cells based on their single-cell expression profiles. The trajectory was visualized using plot_cell_trajectory.

### Cell-cell interaction analysis

Cell-cell interaction analysis was performed using CellPhone DB based on the receptor−ligand interactions between two cell types or subtypes. The cluster labels of all the cells were randomly permuted 1000 times to generate the null distribution of the average ligand-receptor expression levels of the interacting clusters. Individual ligand or receptor expression was assigned a threshold with a cutoff value based on the average log gene expression distribution for all genes across all the cell types. Cell-cell interactions with a p-value < 0.05 and an average log expression > 0.1 were considered significant. These interactions were visualized using the circlize (0.4.10) R package.

### Immunofluorescence

Immunofluorescence staining was performed to achieve the following: verify protein expression, as well as to examine the subcellular localization and co-expression of CD27/CD70 or CXCL13/CXCR5 (**Table 2**). Ocular adnexal MALT lymphoma tissue samples were processed in sequential stages involving deparaffinization, rehydration, and antigen retrieval. For primary antibodies originating from goats, a 10% donkey serum was used as the blocking solution, whereas for primary antibodies from other sources, a 3% BSA solution was used. The tissue were blocked at 37°C for one hour and then incubated with primary antibodies at 4°C overnight. This process was followed by sequential application of HRP-conjugated secondary antibodies and TSA fluorescent dyes, with thorough washing after each step, with the process repeated as appropriate. Finally, the cell nuclei were counterstained with DAPI, followed by the mounting of the samples. The tissue sections were then examined under an inverted microscope and the corresponding images captured for documentation.

**Table 2:** Antibody usage instructions.

Antibody Name and Catalog Number	Manufacturer
Rabbit anti-CXCR5 (Cat#ab254415)	Abcam, United Kingdom
Goat anti-CXCL13 (Cat# AF801-SP)	R&D Systems, United States
Rabbit anti-CD70 (Cat# ab300083)	Abcam, United Kingdom
Goat anti-CD27 (Cat# AF382)	R&D Systems, United States
HRP-conjugated Donkey anti-Goat IgG (Cat# GB23404)	Wuhan Seville Biotech, China
CY3-conjugated Donkey anti-Rabbit IgG (Cat#GB21403)	Wuhan Seville Biotech, China
DAPI Staining Solution (Cat#G1012)	Wuhan Seville Biotech, China

### Statistical analysis

To assess the interaction between cell types, we evaluated the average UMI expression of genes in each cell cluster based on the Pearson correlation. Functional enrichment analysis of DEGs was performed through annotation, visualization, and integration with discovery databases, such as Gene Ontology and KEGG. For differential expression analysis using Seurat, we utilized an unpaired two-tailed Student’s t-test to compare data between two groups. A p-value < 0.05 was considered statistically significant.

## Results

### Cellular diversity and heterogeneity in MALT lymphoma and IgG4-ROD

**Figure 1:**
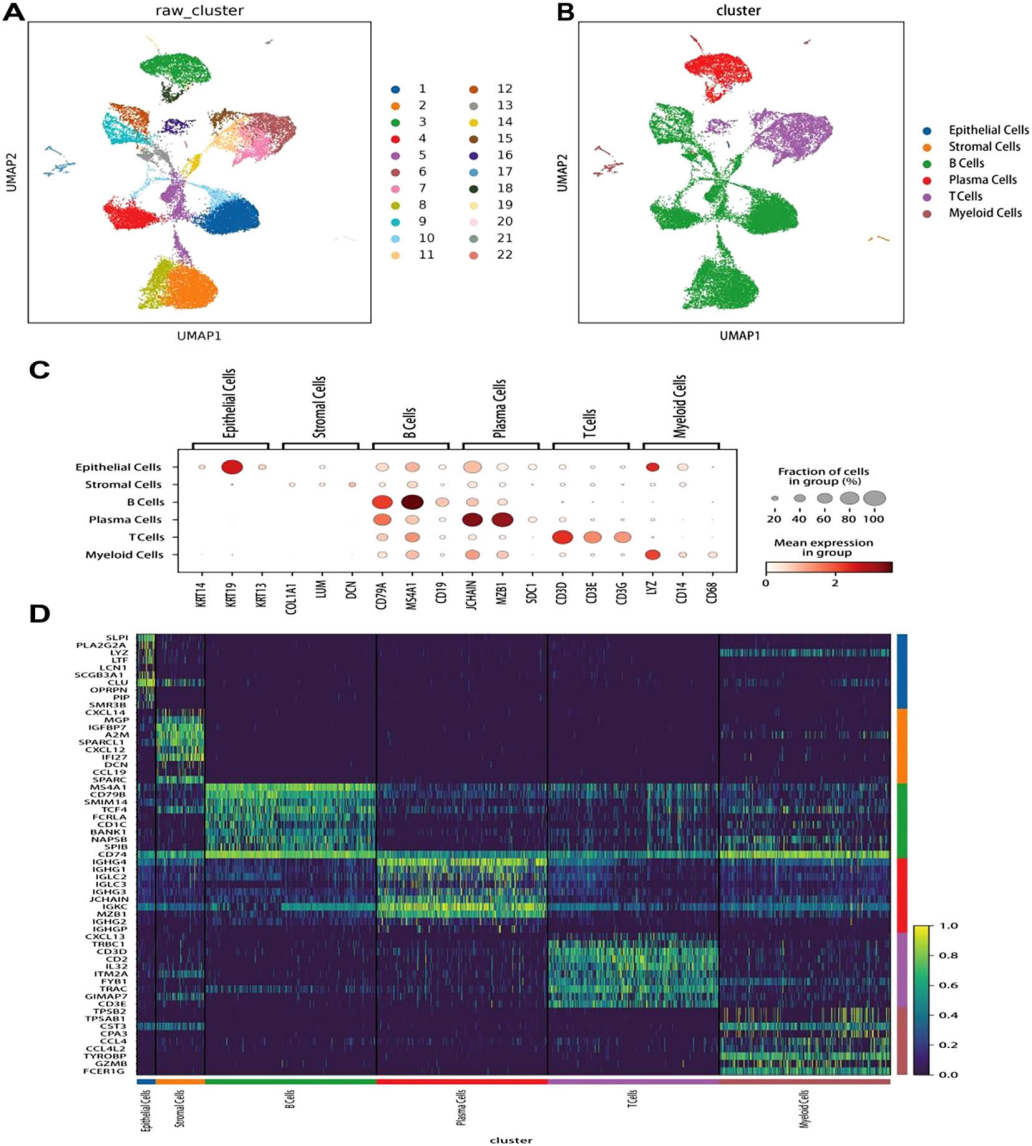
Clustering, cell annotation, and gene expression of all sample data. **(A)** UMAP dimensionality reduction of 22 clusters. Different colors represent different cell clusters, with each dot representing a single cell. **(B)** Classification and annotation results of all cell types, including epithelial, stromal, plasma, and myeloid cells, as well as B- and T-cells. **(C)** Dot plot of the genes with the highest specific gene expression (TOP3) in the cell clusters. **(D)** Heatmap showing the top 10 upregulated genes in each cell type.

In this study, we conducted single-cell expression profiling of three MALT lymphoma and three IgG4-ROD samples. Preprocessing and quality control of the sequencing data were conducted, with the results yielding a dataset of 26,103 single-cell transcriptomes: comprising 17,523 cells from MALT lymphoma tissues and 8,580 cells from IgG4-ROD tissues. The gene expression data was normalized, and then unsupervised clustering method was used leading to the identification of 22 cell clusters (**Figure 1A**). To characterize the cell populations, DEG analysis was conducted between each cluster and all other clusters. We used the cell assignment method to assign these clusters to six distinct cell types based on known marker genes (**Figure 1B**). The cell types and their corresponding marker genes were as follows: B-cells (CD79A, MS4A1, and CD19), T-cells (CD3D, CD3E, and CD3G), plasma cells (JCHAIN, MZB1, and SDC1), stromal cells (COL1A1, LUM, and DCN), myeloid cells (LYZ, CD14, and CD68), and epithelial cells (KRT14, KRT19, and KRT13) (**Figure 1C**). Subsequently, a heatmap of the top 10 genes that were highly expressed in each cell type was generated (**Figure 1D**).

**Figure 2:**
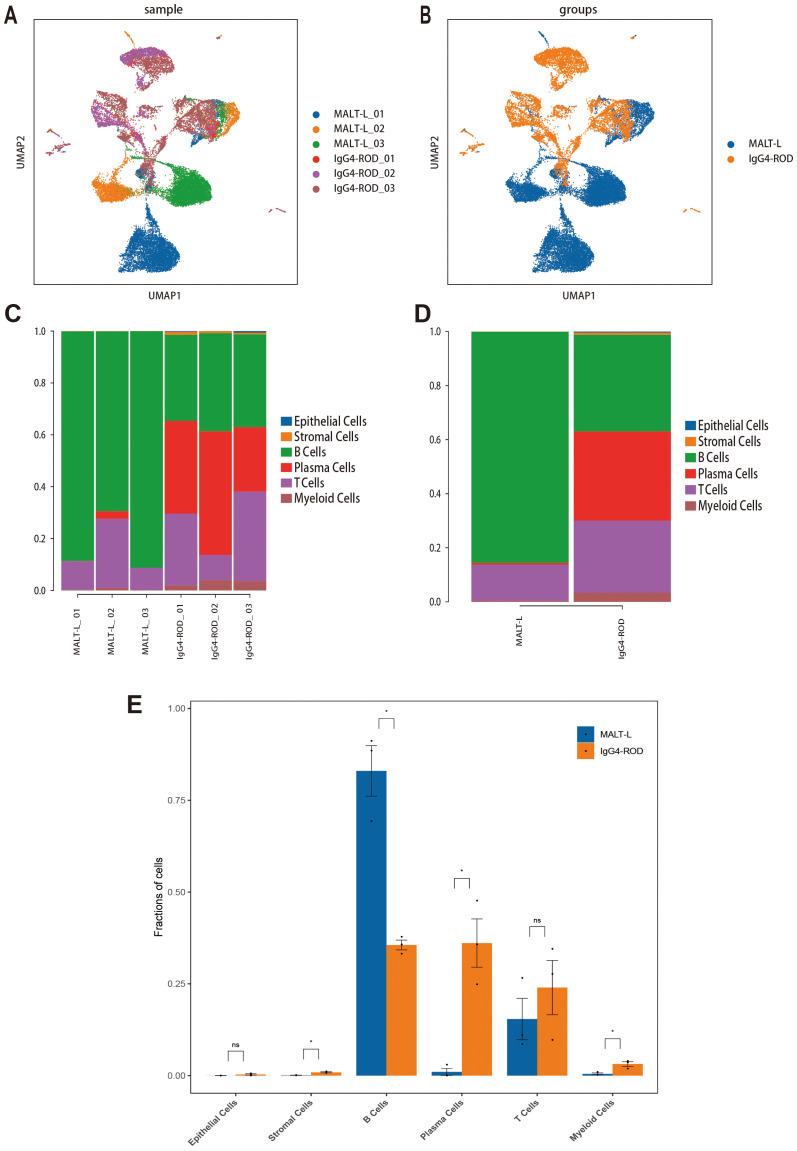
Cell cluster distribution in MALT lymphoma and IgG4-ROD. **(A)** UMAP plots of all cell types in different samples. **(B)** UMAP plot of all cell clusters colored by disease background. **(C)** Percentages of cell type contributions from different sample types. **(D)** Proportion and distribution of all cell types in the two groups. **(E)** Analysis of the significant differences in cell type proportions between the two groups using Student’s t-test. The horizontal axis represents the cell type, the vertical axis represents the average proportion of each cell type, and the color represents the sample group. * indicates a statistically significant difference; ns indicates no statistically significant difference.

We then visualized all the cell types based on their samples and disease group using the UMAP plots (**Figures 2A, B**). Additionally, we compared the relative proportions of each cell type across samples and groups. The results were as follows: in MALT lymphoma tissues, B-cells constituted the majority (85.53%), followed by T-cells (13.37%), while plasma cells (0.64%), myeloid cells (0.38%), and stromal cells (0.09%), with no epithelial cell types in any sample. In the IgG4-ROD tissues, the main subpopulations were B cell (35.78%), plasma cells (33.01%), and T cell (26.64%); myeloid cells (3.37%), stromal cells (0.82%), and epithelial cells (0.38%) accounting for a relatively small proportion (**Figures 2C, D**, **Table 3**). Comparative analysis revealed that the proportion of B-cells in MALT lymphoma patients was significantly greater compared to that in IgG4-ROD patients, whereas the proportions of plasma and stromal cells were significantly greater in IgG4-ROD patients compared to that in MALT lymphoma patients. There were no statistically significant differences in remaining cell types between the two groups (**Figure 2E**).

**Table 3:** Distribution of cell types in patients with MALT lymphoma and IgG4-ROD.

Cell type	MALT lymphoma	Proportion (%)	IgG4-ROD	Proportion (%)
Epithelial cells	0	0	33	0.38
Stromal cells	16	0.09	70	0.82
B cell	14987	85.53	3070	35.78
Plasma cells	112	0.64	2832	33.01
T cell	2342	13.37	2286	26.64
Myeloid cells	66	0.38	289	3.37

### Re-clustering of B-cell subpopulations

**Figure 3:**
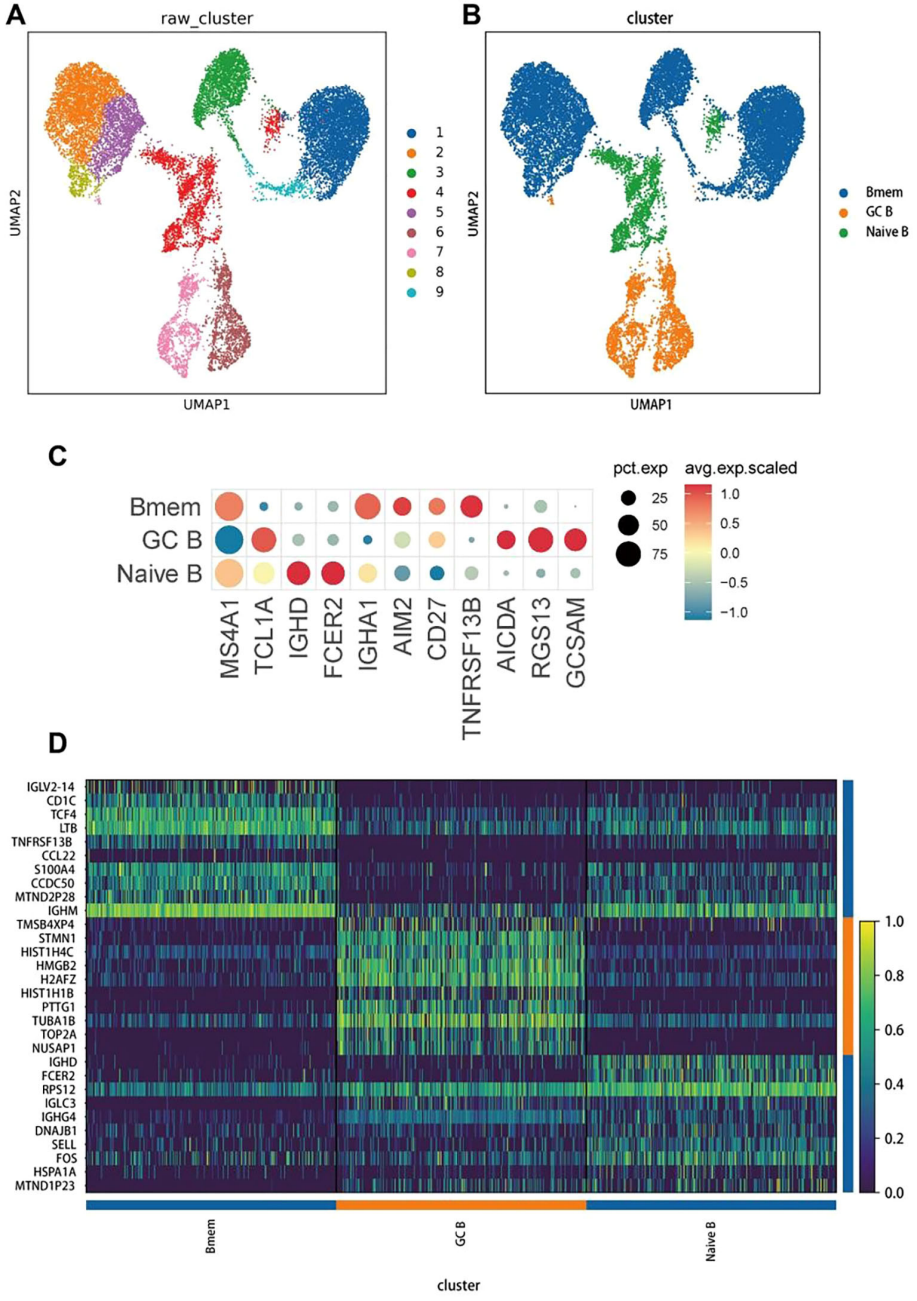
Re-clustering, cell annotation, and gene expression of B cells. **(A)** Results of UMAP dimensionality reduction of the 9 clusters. Different colors refer to different B-cell subsets, with each dot indicating a single cell. **(B)** Classification and annotation analysis of all B-cell subsets, including memory B cells (Bmem), germinal center B cells (GC B), and naive B cells (naive B). **(C)** The dot plot of the genes with the highest specific gene expression (TOP3) of all B-cell subsets. **(D)** A heatmap of the top 10 upregulated genes in each cell type.

To investigate the heterogeneity of B-cells, 18,057 B-cells were re-clustered, resulting in the identification of nine distinct B-cell clusters (**Figure 3A**). Based on the expression of known marker genes in each cell subpopulation, we identified three B-cell subpopulations: memory B (Bmem) cells (markers: IGHA1, AIM2, CD27, and TNFRSF13B), germinal center B (GC B) cells (markers: AICDA, RGS13, and GCSAM), and naive B-cells (markers: MS4A1, TCL1A, IGHD, and FCER2) (**Figures 3B, C**). We then generated a heatmap of the top 10 highly expressed genes in each B-cell subpopulation (**Figure 3D**).

**Figure 4:**
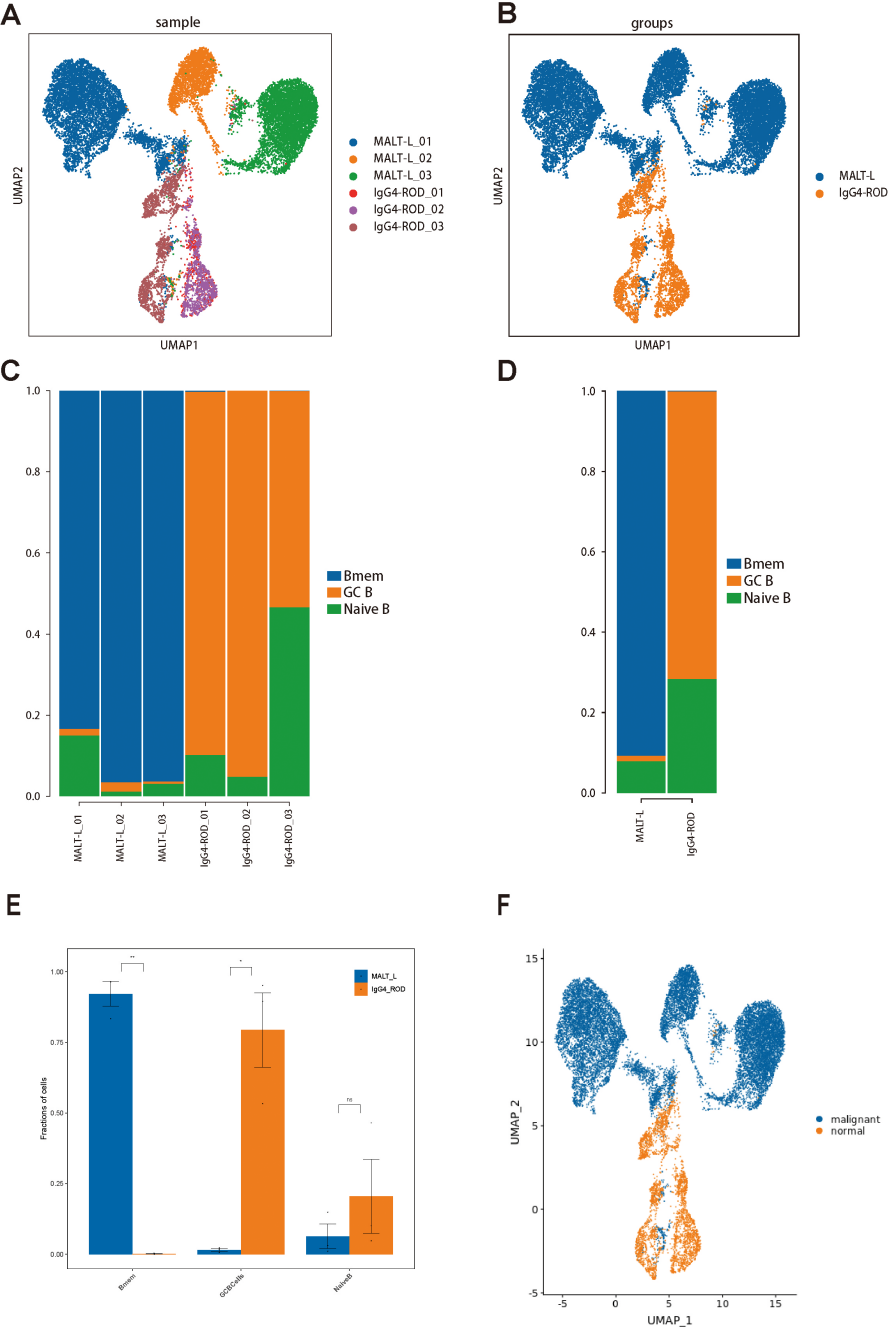
B-cell subset distributions in MALT lymphoma and IgG4-ROD patients. **(A)** Representative UMAP plots of all B-cell subsets in different samples. **(B)** UMAP plot of all cells colored based on the disease background. **(C)** Percentages of cell type contributions from different sample types. **(D)** The proportion and distribution of all B-cell subsets in the two groups. **(E)** Comparison of cell type proportions between the two groups (t test). The horizontal axis represents the cell types, the vertical axis represents the average proportion of each cell type, and the color reflects the sample groups. **(F)** UMAP plot illustrating the benign and malignant B-cell subpopulations. * indicates a statistically significant difference; ns indicates no statistically significant difference.

We then generated UMAP plots of B-cell subpopulations based on the sample classification or disease group (**Figures 4A, B**). Subsequently, we compared the relative proportions of the three B-cell subpopulations across samples and groups. The results were as follows: in the MALT lymphoma tissues, the Bmem cell subpopulation was predominant, followed by the naive B-cell subpopulation, and then the GC B-cell subpopulation being the least represented; Conversely, in the IgG4-ROD tissues, the GC B-cell subpopulation was predominant, followed by the naive B-cell subpopulation, with the Bmem cell subpopulation being the least represented (**Figures 4C, D**). Comparative analysis revealed that the B-cell subpopulation in MALT lymphoma patients was primarily Bmem cells, whereas the B-cell subpopulation in IgG4-ROD patients was mainly GC B cells (**Figure 4E**).

### Inter-transcriptomic heterogeneity of malignant B-cells in MALT lymphoma

**Figure 5:**
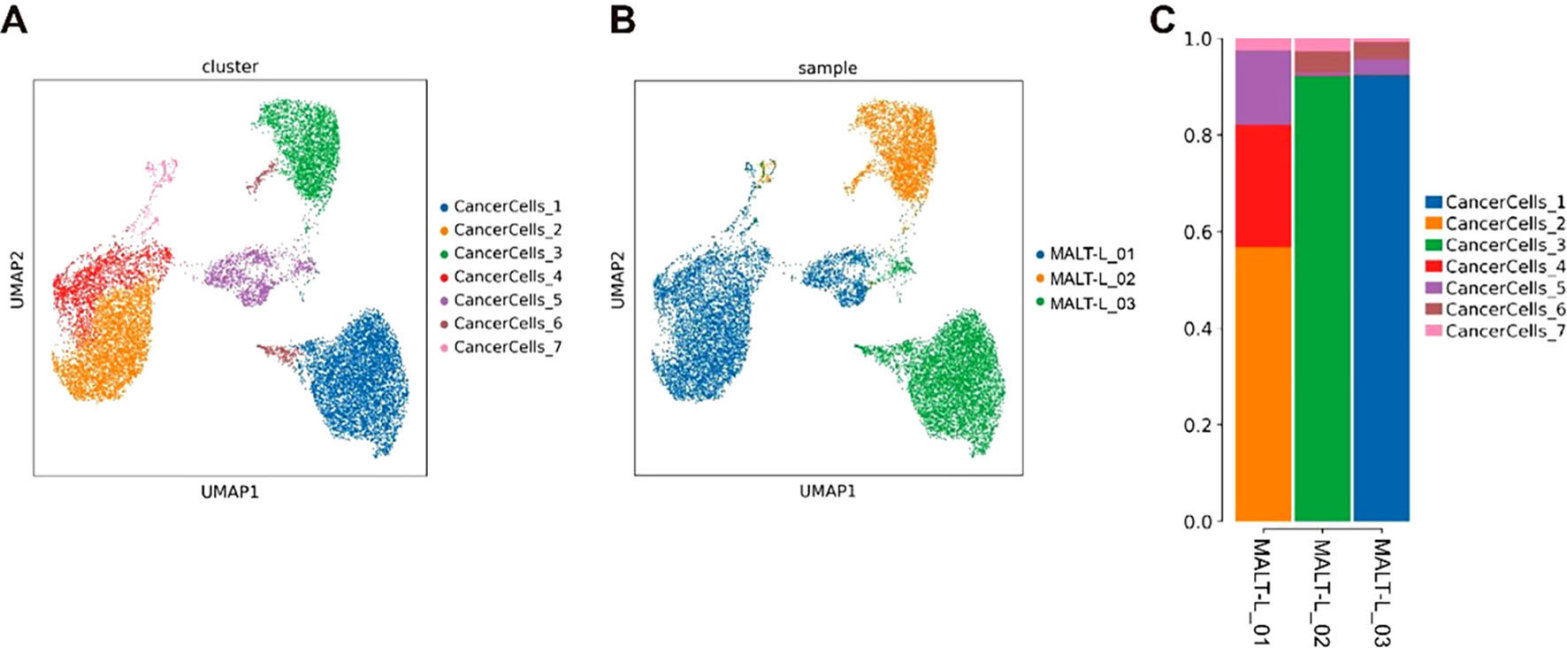
Heterogeneity of malignant B cells in MALT lymphoma. **(A)** The UMAP plot colored by cluster, showing 7 tumor cell subpopulations. **(B)** UMAP plot colored based on the sample, showing that the distribution of tumor cell subpopulations in each sample is relatively dispersed. **(C)** Bar chart of the cell cluster composition of individual samples, showing that different samples have specific tumor cell subpopulations.

To distinguish malignant and nonmalignant B-cells, we observed the expression of malignant B-cell population in a specific type of immunoglobulin light chain, κ or λ light chains. The ratio of light chains per B-cell (κ/λ) was calculated depending on the expression of the genes IGKC (encoding a constant portion of the κ light chain) and IGLC2 λ light chain). Ocular adnexal MALT lymphomas contain malignant B-cells that uniformly express either κ or λ light chains, whereas IgG4-ROD samples contain only nonmalignant B-cells (**Figure 4F**). Subsequently, we re-clustered the malignant B-cells and obtained seven malignant B-cell subpopulations (**Figure 5A**), representing a high degree of heterogeneity (**Figures 5B, C**).

### Re-clustering of T-cell subpopulations

**Figure 6:**
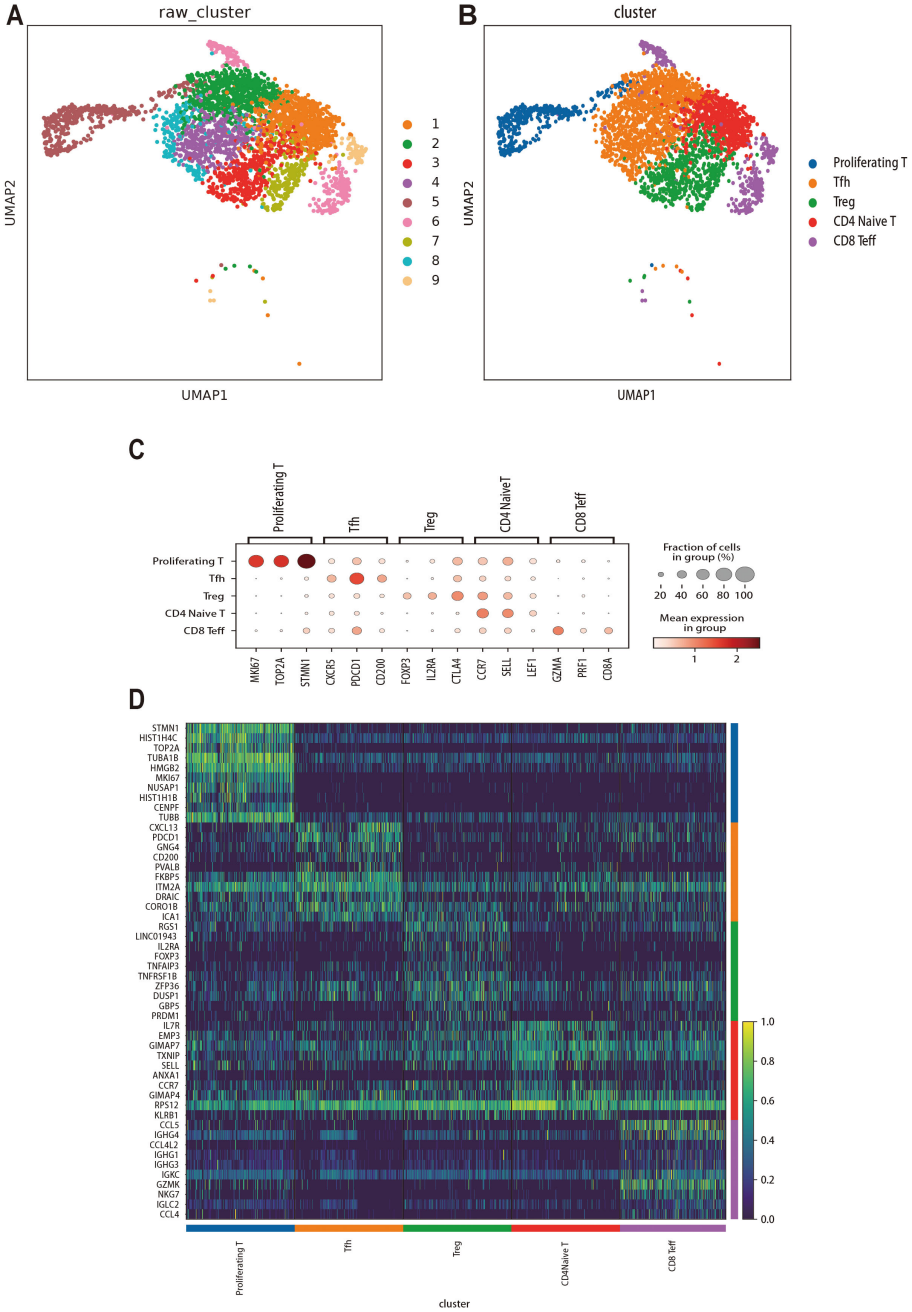
Re-clustering, cell annotation, and gene expression analysis of T cells in MALT lymphoma and IgG4-ROD. **(A)** UMAP dimensionality reduction of 9 clusters. The colors reflect the different T-cell subsets, with each dot indicating a single cell. **(B)** Classification and annotation results of all T-cell subsets, such as proliferating T cells, follicular helper T (Tfh) cells, regulatory T (Treg) cells, CD4+ naive T cells, and CD8+ effector T (CD8 Teff) cells. **(C)** Dot plot for the genes with the highest specific gene expression (top 3) of all T-cell subsets. **(D)** Heatmap of the top 10 upregulated genes in each cell type.

To investigate the transcriptomic heterogeneity of T-cells, we re-clustered 3309 T cells (1662 from MALT lymphoma samples and 1647 from IgG4_ROD samples) and obtained the nine distinct clusters (**Figure 6A**). Based on the expression patterns of marker genes in each cell subgroup, we identified five T-cell subpopulations: CD8+ effector T (CD8 Teff) cells (marker genes GZMA, PRF1, and CD8A), regulatory T (Treg) cells (marker genes FOXP3, IL2RA, and CTLA4), follicular helper T (Tfh) cells (marker genes CXCR5, PDCD1, and CD200), proliferating T-cells (marker genes MKI67, TOP2A, and STMN1), and CD4+ naive T-cells (marker genes CCR7, SELL, and LEF1) (**Figures 6B, C**). Each T-cell subpopulation displayed distinct profiles of highly expressed genes. Subsequently, we generated a heatmap to visualize the top 10 highly expressed genes within each cell type (**Figure 6D**).

**Figure 7:**
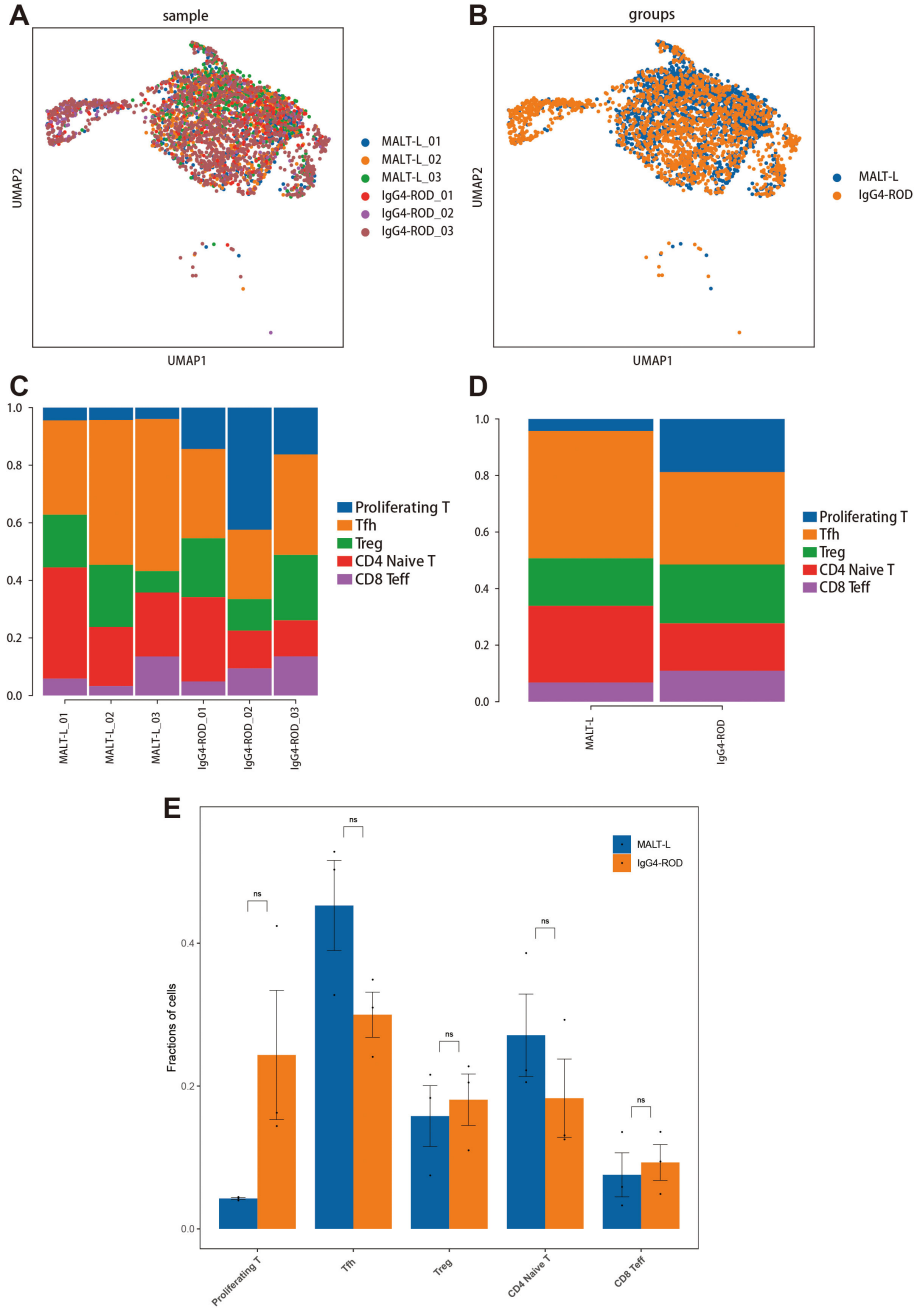
All T-cell subcluster distributions in MALT lymphoma and IgG4-ROD. **(A)** Representative UMAP plots of all T-cell subpopulations in the indicated samples. **(B)** The UMAP plot of all T-cell subpopulation clusters colored by disease background. **(C)** Percentages of all T-cell subpopulation contributions from different sample types. **(D)** The proportions and distributions of all T-cell subpopulations in the two groups. **(E)** Analysis of the significant differences in T-cell subpopulation proportions between the two groups (t-test). The horizontal axis indicates the cell types, the vertical axis reflects the average proportion of each cell type, and the color represents the sample group. ns indicates no statistically significant difference.

We used UMAP plots to visualize all T-cell subpopulations categorized by disease and sample (**Figures 7A, B**). Additionally, we compared the relative proportions of the five T-cell subpopulations across the samples and groups. The results indicated that the proportions of Tfh cells and CD4+ naive T-cells were greater in MALT lymphoma tissues, whereas the proportions of proliferating T-cells, Treg cells, and CD8+ Teff cells were relatively greater in IgG4-ROD tissues (**Figures 7C, D**). Notably, these differences were not statistically significant (**Figure 7E**).

### Functional analysis of DEGs in T cell subpopulations in MALT lymphoma and IgG4-ROD

**Figure 8:**
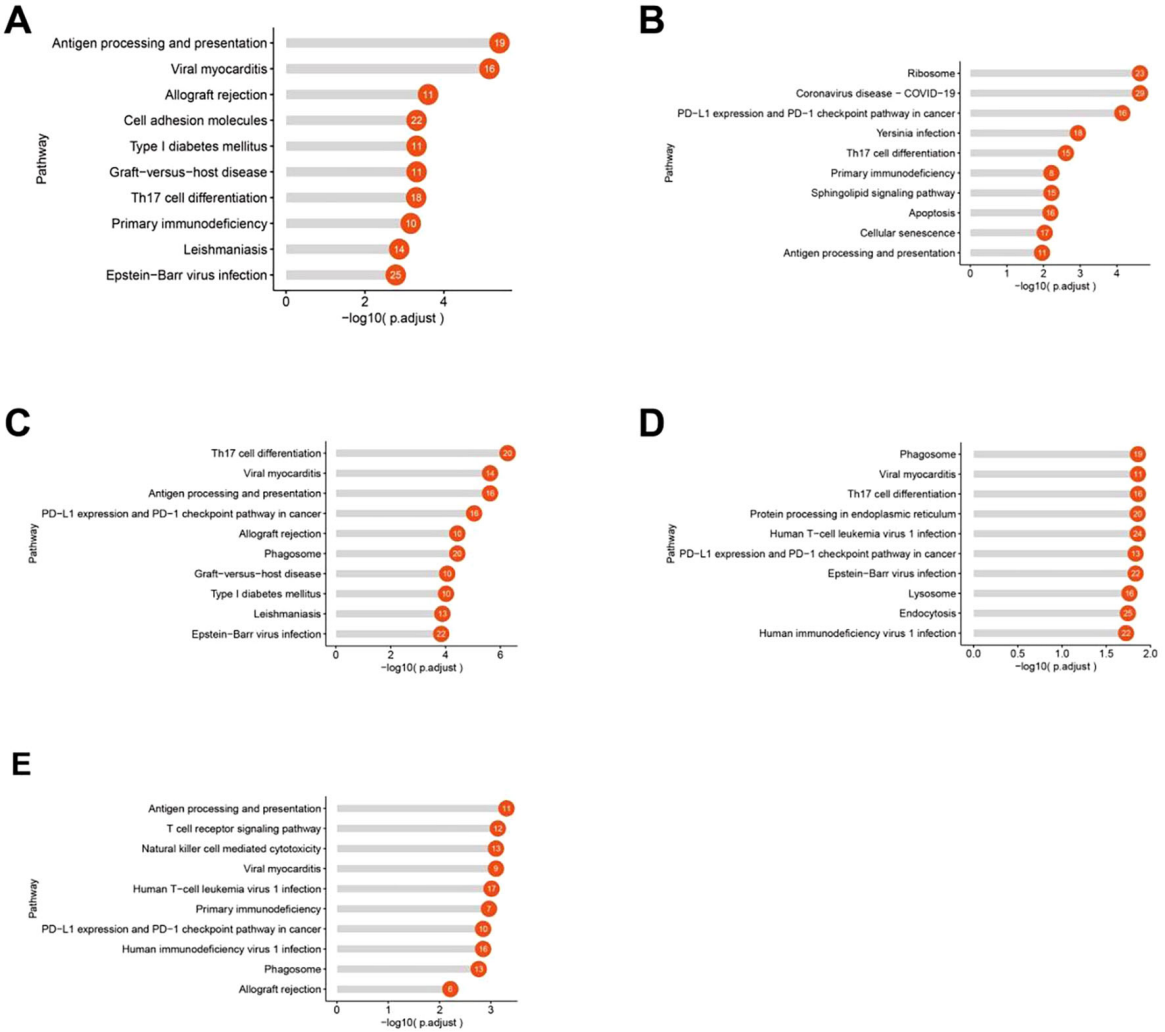
KEGG pathway enrichment analysis of upregulated genes in each T-cell subtype in the MALT lymphoma (compared with the IgG4-ROD). **(A)** Significantly enriched pathways associated with upregulated genes in proliferating T cells in MALT lymphoma. **(B)** The enriched pathways associated with upregulated genes in CD4+ naive T cells in MALT lymphoma. **(C)** Significantly enriched pathways associated with genes upregulated in Tfh cells in MALT lymphoma. **(D)** Significantly enriched pathways associated with genes upregulated in Treg cells in MALT lymphoma. **(E)** Significantly enriched pathways associated with genes upregulated in CD8+ effector T cells (CD8+ Teffs) in MALT lymphoma.

To explore the functional differences between T-cell subpopulations in MALT lymphoma and IgG4-ROD patients, we conducted KEGG pathway enrichment analysis of the DEGs between the two groups. The results revealed distinct enrichment patterns:

Proliferating T-cells: Upregulated genes were significantly enriched in pathways associated with antigen processing and presentation, cell adhesion, and Th17 cell differentiation (**Figure 8A**). CD4+ naive T-cells: Upregulated genes in MALT lymphoma were significantly enriched in pathways associated with ribosome function, PD-1 and PD-L1 signaling, Th17 cell differentiation, and antigen presentation pathways (**Figure 8B**). Tfh cell: Upregulated genes were significantly enriched in pathways associated with Th17 cell differentiation, antigen processing and presentation, and PD-1 and PD-L1 signaling (**Figure 8C**). Treg cells: The upregulated genes were enriched in pathways associated with Th17 cell differentiation, protein processing in the endoplasmic reticulum, and PD-1 and PD-L1 signaling (**Figure 8D**). CD8+ Teff cells: Enriched pathways included antigen processing and presentation, T-cell receptor signaling, NK cell-mediated cytotoxicity, and the PD-1 and PD-L1 signaling pathways (**Figure 8E**).

These findings highlight the various functional roles of T-cell subpopulations in MALT lymphoma and IgG4-ROD, underscoring potential differences in immune regulation and tumor microenvironment (TME) interactions.

### Pseudotime trajectory analysis of three CD4+ T-cell subpopulations

**Figure 9:**
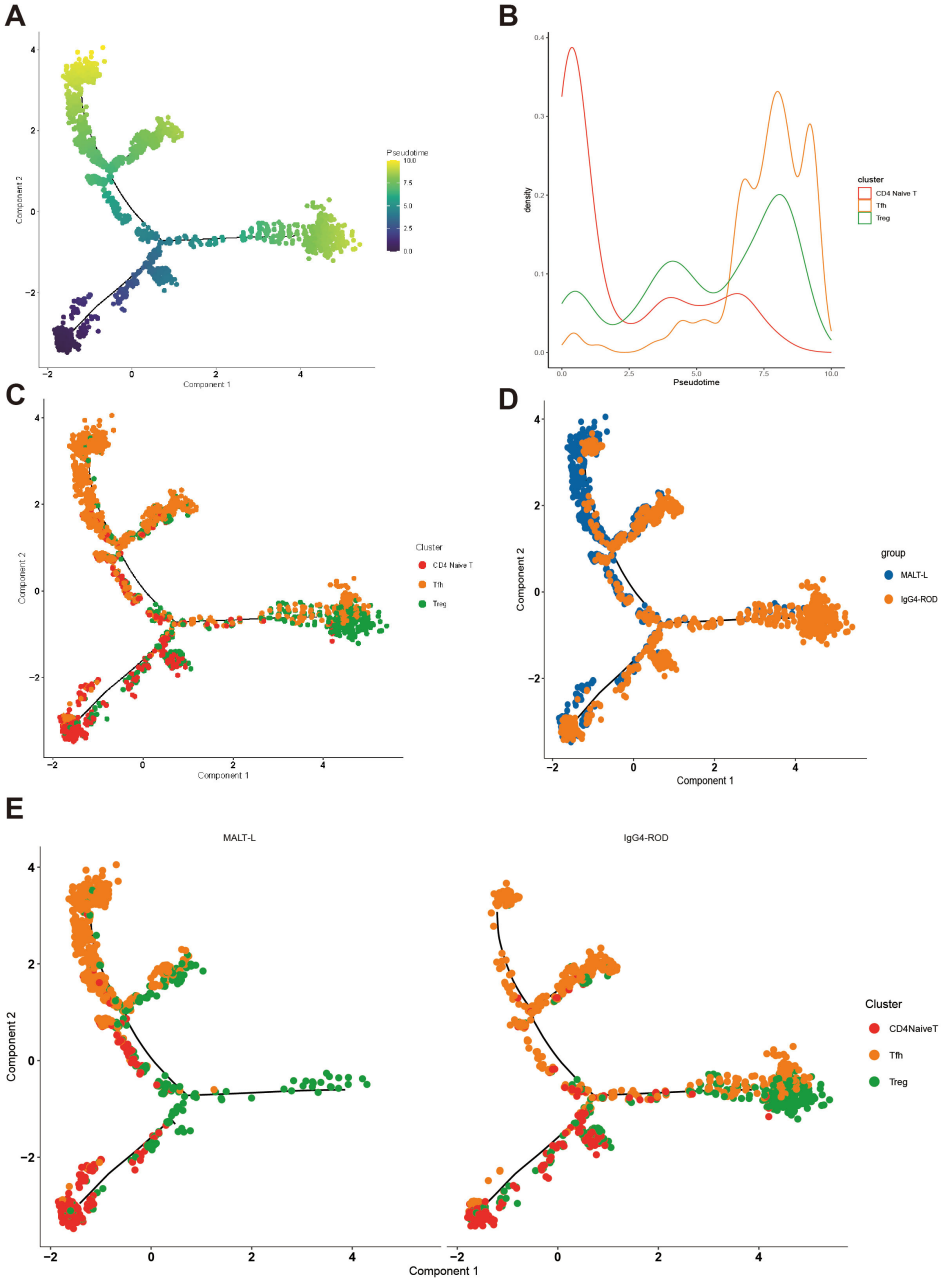
Pseudotime trajectory analysis of three CD4+ T-cell subpopulations. **(A)** Pseudotime trajectory analysis of CD4+ naive T cells, follicular helper T cells, and regulatory T cells. The order of time refers to the order of cell differentiation. **(B)** Analysis of the density distribution of different cell types over time. The horizontal coordinate indicates the pseudotime coordinate, and the vertical coordinate represents the density of cell numbers in the indicated time-points. **(C)** Pseudotime trajectory analysis of the different cell types in a graph reflecting the differentiation relationships of the indicated cell types. **(D)** Pseudotime trajectory analysis of grouped coloring maps by disease type, with different colors representing different subgroups. **(E)** The distribution of pseudotime trajectory analysis of different cell types in each disease group, with different colors representing different cell types.

Our findings have demonstrated that CD4+ T-cells play a crucial role in promoting the growth of MALT lymphoma. Subsequently, pseudotime analysis was conducted on CD4+ naive T, Treg, and Tfh cell subpopulations using the Monocle 2 method to investigate the differentiation trajectory of CD4+ T-cells. We constructed a tree-like branching structure diagram depicting the differentiation trajectories of three CD4+ T-cell subpopulations (**Figures 9A, B**). From a cellular perspective, differentiation predominantly initiates from CD4+ naive T-cells, with two distinct branches representing Tfh and Treg cells (**Figure 9C**). This observation is consistent with the biological process of CD4+ T-cell differentiation. Comparative analysis revealed that in MALT lymphoma, CD4+ naive T-cells predominantly differentiated into Tfh cells, whereas in IgG4-ROD lymphoma, they mainly differentiated into Treg cells (**Figures 9D, E**). This corroborates the finding that the proportion of Tfh cells is greater in MALT lymphoma tissues, whereas the proportion of Treg cells is relatively greater in IgG4-ROD tissues.

### Cellular communication between malignant B-cells and T-cell subpopulations in MALT lymphoma

**Figure 10:**
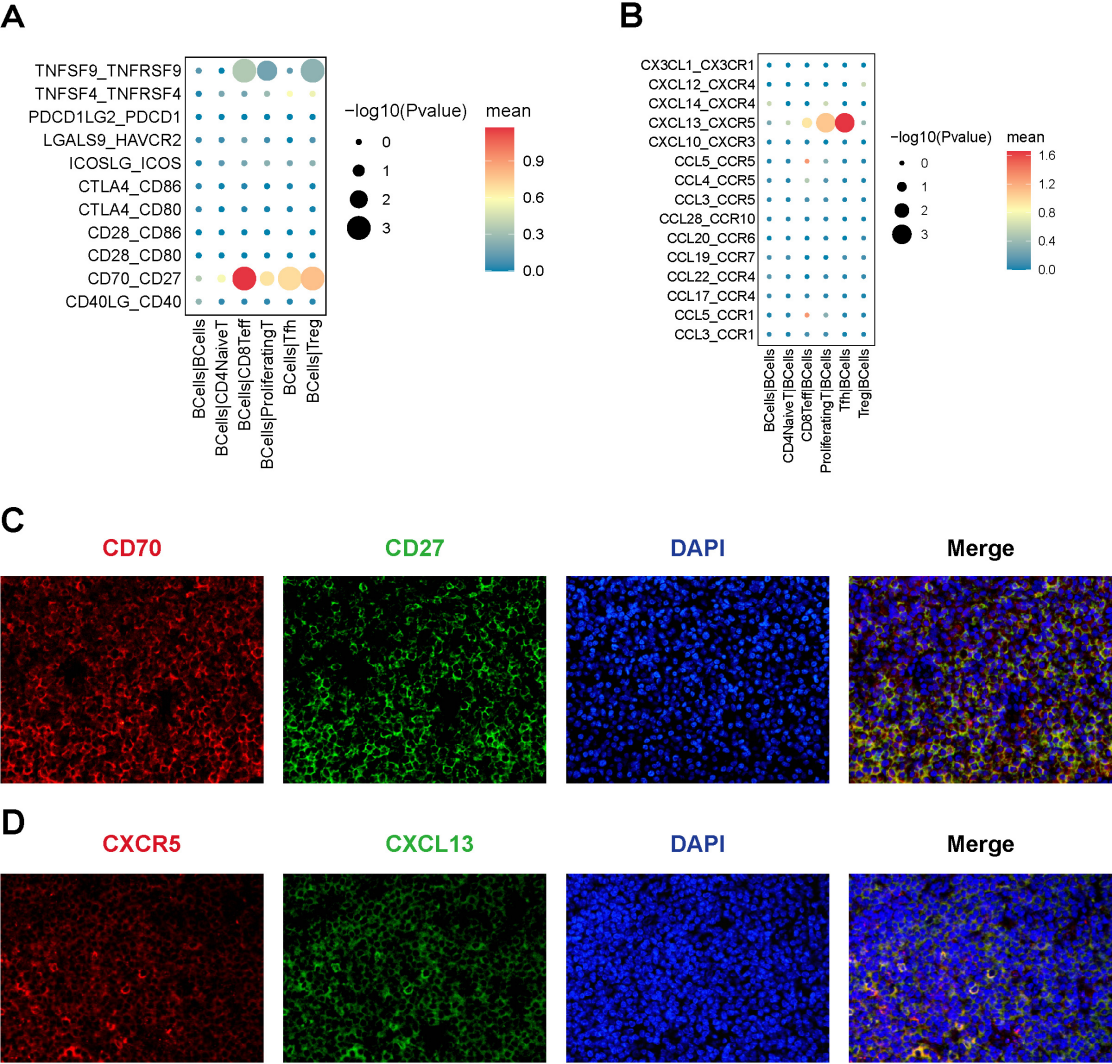
Cellular interactions between B-cell and T-cell subpopulations. **(A)** The Bubble chart of immune checkpoint interactions, with B cells as ligand cells and the predicted immune checkpoint interaction pairs between B cells and T-cell subtypes; **(B)** The Bubble chart of chemokine interaction pairs, with B cells as receptor cells and the predicted chemokine interaction pairs between B cells and T-cell subtypes. **(C)** Results of the immunofluorescence staining of CD70-CD27 (x200), DAPI staining (x200), and merged image (x200); **(D)** Results of the immunofluorescence staining of CXCR5-CXCL13 (x200), DAPI staining (x200), and merged image (x200).

Notably, B-cells and T-cells are the primary cell types involved in ocular adnexal MALT lymphoma. Subsequently, we focused on analyzing the cellular communication between B-cell and T-cell subpopulations. We examined receptor-ligand pairs associated with immune checkpoints and chemokine, resulting in the identification of several potential receptor-ligand interactions that facilitate the progression of MALT lymphoma. These pairings were validated using immunofluorescence assays. Our findings revealed the following:

CD70-CD27: We observed that tumor B-cells expressed high levels of CD70, which interact with CD27 to provide coinhibitory signals to CD8+ Teff cells (**Figure 10A**). This interaction promotes the exhaustion and inhibition of CD8+ Teff cells, thereby facilitating immune evasion in lymphoma. Immunofluorescence double staining technique showed the co-expression of CD70 and CD27 in MALT lymphoma samples (**Figure 10C**).

CXCL13-CXCR5: Tfh cells highly expressed CXCL13, recruiting tumor B-cells through the CXCL13-CXCR5 interaction (**Figure 10B**). Immunofluorescence double staining showed the co-expression of CXCL13 and CXCR5 in MALT lymphoma samples (**Figure 10D**).

## Discussion

In this study, we conducted a comprehensive analysis of the single-cell transcriptomes of MALT lymphoma and IgG4-ROD patients. The results revealed distinct differences in the cellular subpopulation composition and transcriptional heterogeneity between the two conditions. Specifically, all B-cells in MALT lymphoma are malignant and display significant intra- and intertumoral heterogeneity. We then conducted cell communication analyses between malignant B-cell and T-cell subpopulations and identified several receptor-ligand interactions that play crucial roles in the development and progression of MALT lymphoma. This observation was validated using immunofluorescence double-staining assays. Our findings provide vital insights into the cellular composition, essential pathways, and key immune microenvironment characteristics involved in the progression of ocular adnexal MALT lymphoma and IgG4-ROD.

To characterize the cellular composition of MALT lymphoma and IgG4-ROD, we conducted expression profiling on 26,103 cells derived from the three MALT and three IgG4-ROD samples. We conducted a clustering analysis and identified six distinct cellular subpopulations: B-cells, T-cells, plasma cells, stromal cells, myeloid cells, and epithelial cells. Through comparative analysis, we observed a significant expression level of B-cells in MALT lymphoma patients compared to IgG4-ROD patients. Conversely, plasma and stromal cells were more significantly expressed in IgG4-ROD patients. These observations are consistent with the histopathological characteristics of these diseases (10, 23). In MALT lymphoma, the clonal proliferation of small B-cells results in a significant increase in the proportion of B-cells. Conversely, in IgG4-ROD, significant pathological features, including plasma cell infiltration and substantial tissue fibrosis – observed in some patients contributing to the increased proportion of plasma and stromal cells.

Re-clustering analysis of the B-cell subpopulations revealed significant number of memB cells in MALT lymphoma patients and a substantial number of GC B-cells in IgG4-ROD patients. This finding corroborates the claim that MALT lymphoma originates from memory B-cells, as well as validating the histopathological observation of GC B-cells in IgG4-ROD (23–25). Memory B-cells, characterized by their enhanced antigen recall capabilities, represent the persistent immune response in MALT lymphoma. Germinal center B-cells, which play a significant role in antibody affinity maturation and immune regulation, are likely contributors to the inflammation and immune dysregulation in IgG4-ROD.

Malignant B-cell proliferation is a hallmark of MALT lymphoma. Based on the κ or λ light chain restrictions, a prominent characteristic of malignant lymphocytes, we found that all B-cells in MALT lymphoma exhibit malignant characteristics, whereas B-cells in IgG4-ROD are nonmalignant. This finding aligns with the malignant nature of MALT lymphoma and benign inflammatory characteristic of IgG4-ROD (18). Upon further analysis, a significant degree of intra- and intertumoral heterogeneity was observed among malignant B-cells in MALT lymphoma.

The immune microenvironment of MALT lymphoma and IgG4-ROD is predominantly comprised of T-cells. We identified five distinct T-cell subpopulations: CD8+ Teff, Tfh, Treg, proliferating T, and CD4+ naive. Through pseudotime analysis, we observed that naive CD4+ T-cells differentiated into two branches, namely Tfh and Treg cells. Through comparative analysis, we observed that naive CD4+ T-cells predominantly differentiate into Tfh cells in MALT lymphoma, whereas in IgG4-ROD, they are more likely to differentiate into Treg cells. This differentiation bias resulted in a greater proportion of Tfh cells in MALT lymphoma compared to that in IgG4-ROD. These findings suggest that Tfh cells play a crucial role in the pathogenesis and progression of MALT lymphoma. Previous studies have shown that Tfh cells are present in the immune microenvironments of various lymphomas, including diffuse large B-cell and marginal zone lymphomas, significantly contributing to lymphoma development. Monitoring Tfh cells can provide prognostic insights for lymphoma patients (26, 27). Therefore, therapeutic strategies aimed at targeting Tfh cells hold significant clinical value and present promising potential for improving treatment outcomes.

Intergroup differential gene KEGG pathway enrichment results indicated that both the CD4+ T-cell and CD8+ Teff cell subpopulations in MALT lymphoma patients were significantly enriched in the PD-1/PD-L1 pathway, compared with those in IgG4-ROD patients. These findings suggest that these T-cell subpopulations play crucial roles in promoting tumor growth and immune evasion via the PD-1/PD-L1 pathway. Currently, research has revealed that immunotherapies involving PD-1 or PD-L1 blockade have superior efficacy in the treatment of various cancers, including non-small cell lung cancer (28), melanoma (29), breast cancer (30), ovarian cancer (31), and classical Hodgkin lymphoma (32). However, studies focusing on the mechanism of the PD-1/PD-L1 pathway in the management of ocular adnexal MALT lymphoma remain scarce. Our findings indicate that a deeper investigation into the role of this pathway in MALT lymphoma holds a significant scientific value and potential clinical implications.

The initiation and progression of tumors are significantly influenced by the complex interactions among various cells within the TME (33). These cells are engaged in a complex interaction network through multiple cell-to-cell communication mechanisms, such as ligand-receptor interactions, paracrine and autocrine signaling, consequently influencing tumor development (34). We focused on the costimulatory and coinhibitory interactions between malignant B-cells and various T-cell subpopulations within the MALT TME. Our results implied a potential connection between the CD70-CD27 signaling axis and the development of MALT lymphoma. Additionally, recent studies have emphasized the crucial role of the CD70−CD27 signaling pathway-mediated immune evasion mechanism in the development and progression of various lymphomas, including Diffuse Large B-Cell Lymphoma, Mantle Cell Lymphoma, and T-cell lymphoma (35–37). Therefore, blocking the CD70/CD27 signaling axis represents a novel approach in lymphoma immunotherapy.

Given the lack of germinal center-containing lymphoid tissues within the ocular adnexal structures, potentially, the etiology of MALT lymphoma involves lymphocyte migration and localization (10). Chemokines play a pivotal role in this process; hence, we investigated the distribution of chemokine-related ligand-receptor pairs across different cellular subpopulations. Our research pointed that the CXCL13-CXCR5 signaling pathway may play a role in the development of MALT lymphoma. This interaction plays a significant role in the pathophysiology of MALT lymphoma, a finding that aligns with previous research that has reported the expression of CXCL13 and CXCR5 in ocular adnexal MALT lymphoma, suggesting their potential role in mediating lymphocyte homing and retention within the ocular adnexa (38, 39). Consequently, targeting the CXCL13/CXCR5 signaling axis represents a promising immunotherapeutic approach for managing and treating ocular adnexal MALT lymphoma.

### Limitations of the study

There was no data from the control sample in this study, which is due to the limited number of normal ocular mucosa-associated lymphoid tissues. This limitation hinders the comparison of data from both the normal and pathological tissues, which could provide vital insights into the disease. Another limitation of this study is the absence of subsequent investigations to validate our findings. Future research should focus on substantiating these findings through comprehensive molecular biology, cytological, and animal model experiments to ensure their validity and broader applicability.

## Data Availability

The raw sequence data reported in this paper have been deposited in the Genome Sequence Archive (Genomics, Proteomics & Bioinformatics 2021) in National Genomics Data Center (Nucleic Acids Res 2022), China National Center for Bioinformation / Beijing Institute of Genomics, Chinese Academy of Sciences (GSA-Human: HRA010437) that are publicly accessible at https://ngdc.cncb.ac.cn/gsa-human.
